# PEG-crosslinked O-carboxymethyl chitosan films with degradability and antibacterial activity for food packaging

**DOI:** 10.1038/s41598-024-61642-x

**Published:** 2024-05-11

**Authors:** Bing Yang, Baoliang Liu, Yuanyuan Gao, Junjie Wei, Gang Li, Hui Zhang, Linlin Wang, Zhaosheng Hou

**Affiliations:** 1 Key Laboratory of Public Security Management Technology in Universities of Shandong, School of Intelligence Engineering, Shandong Management University, Jinan, Shandong China; 2 School of Chemistry and Chemical Engineering, Qilu Normal University, Jinan, Shandong China; 3 Taian Yingxiongshan Middle School, Taian, Shandong China; 4 Shandong Tianming Pharmaceutical Co, Ltd., Jinan, Shandong China; 5 College of Chemistry, Chemical Engineering and Materials Science, Shandong Normal University, Jinan, Shandong China

**Keywords:** O-Carboxymethyl chitosan, Polyethylene glycol, Crosslinking, Degradable, Antibacterial activity, Materials chemistry, Sustainability

## Abstract

This study developed a kind of PEG-crosslinked O-carboxymethyl chitosan (O-CMC–PEG) with various PEG content for food packaging. The crosslinking agent of isocyanate-terminated PEG was firstly synthesized by a simple condensation reaction between PEG and excess diisocyanate, then the crosslink between O-carboxymethyl chitosan (O-CMC) and crosslinking agent occurred under mild conditions to produce O-CMC–PEG with a crosslinked structure linked by urea bonds. FT-IR and ^1^H NMR techniques were utilized to confirm the chemical structures of the crosslinking agent and O-CMC–PEGs. Extensive research was conducted to investigate the impact of the PEG content (or crosslinking degree) on the physicochemical characteristics of the casted O-CMC–PEG films. The results illuminated that crosslinking and components compatibility could improve their tensile features and water vapor barrier performance, while high PEG content played the inverse effects due to the microphase separation between PEG and O-CMC segments. The in vitro degradation rate and water sensitivity primarily depended on the crosslinking degree in comparison with the PEG content. Furthermore, caused by the remaining –NH_2_ groups of O-CMC, the films demonstrated antibacterial activity against *Escherichia coli* and *Staphylococcus aureus*. When the PEG content was 6% (medium crosslinking degree), the prepared O-CMC–PEG_−6%_ film possessed optimal tensile features, high water resistance, appropriate degradation rate, low water vapor transmission rate and fine broad-spectrum antibacterial capacity, manifesting a great potential for application in food packaging to extend the shelf life.

## Introduction

Nowadays, the food safety problems caused by food contamination have become one of the most prominent public health problems around the world with the accelerated global food circulation. Approximately 30% of the global population experience serious health problems through consumption of food containing pathogens^1^. Furthermore, foods, especially fruits and vegetables, bring more security problems because they are perishable in the process of storage, transportation, and sale. Appropriate packaging material can maintain food quality and avoid possible contamination along the entire food supply chain^2^. Various petroleum-based packaging systems such as PP and PE have been widely used in our daily lives^3,4^, while these materials face a serious problem of environmental pollution because of their non-degradable nature^5^. Therefore, natural polymer materials have been considered as promising candidates to develop food packaging materials because of their non-toxicity, renewability, low cost, good biocompatibility, degradability, and high consumer acceptance^6–9^.

As a natural biopolymer material, chitosan (CS), the most abundant alkaline polysaccharide in nature^10^, has been extensively used in food, biomedicine, and other aspects due to its diverse bioactivities, such as biocompatibility, degradability, non-toxicity, and antibacterial activity^11–14^. As expected, CS has also drawn attention as an ideal environmental material regarding the replacement of petroleum-based materials^15^ and is becoming one of the key raw materials for preparing antibacterial packaging materials^16,17^. Despite the promise, strong intermolecular H-bonds existing in the crystalline structure of CS produced poor tensile properties^18^. Additionally, CS has limited solubility in water and most organic solvents, further restricting its application in packaging^19–21^. O-carboxymethyl chitosan (O-CMC), a derivative of CS, possesses similar chemical properties but offers improved water solubility, superior antibacterial activity, and enhanced chemical activity due to the introduction of the carboxymethyl group^22–25^. However, O-CMC has also drawbacks such as brittleness and weak gas permeability, which hinder its application in the field of food packaging^26,27^. Modification of O-CMC can improve the tensile properties, gas barrier properties, and so on, so considerable work has been carried out to promote its properties of O-CMC^28,29^.

Polyethylene glycol (PEG) is a biocompatible and non-toxic polyether that can dissolve in water and various organic solvents without undergoing hydrolysis^30–33^. The double-ended hydroxyl (–OH) structure of PEG allows for modifications with functional groups like carboxyl, hydroxyl, and amides^34^. As a result, PEG has been widely used as a modifier to enhance the properties of materials, including plasticity, hydrophilicity, and flexibility^35^. In a recent work^36^, we reported a class of monomethoxyl PEG grafted O-CMC films, which simultaneously exhibited excellent tensile properties, satisfactory water vapor barrier properties, and good broad-spectrum antibacterial activities. The nontoxic materials hold significant potential for edible food packaging. However, the application range of the composites was greatly limited by their high water solubility.

To solve the limitations of the above CS-based film materials, a novel food packaging material was designed with a simple strategy in this study. The condensation reaction between PEG and excess diisocyanate readily accomplished to produce NCO-terminated PEG (OCN–PEG–NCO), which was used as a crosslinking agent to react with O-CMC in water under mild conditions to produce PEG-modified O-CMC (O-CMC–PEG) with crosslinked structure linked by urea bonds. The influences of the PEG content (or crosslinking degree) on the physicochemical features (thermal properties, tensile properties, water sensitivity, water vapor barrier properties, degradability) of the casted O-CMC–PEG films were extensively investigated. In addition, the antibacterial performance of the composite film was evaluated.

## Materials and methods

### Materials

O-CMC (M_W_: 240 KDa, substitution degree: 90%; deacetylation degree: 90%) and PEG (M_n_: 1000 g/mol, dried for 150 min at 100 °C under vacuum) were acquired from Sigma-Aldrich (Shanghai, China). Hexamethylene diisocyanate (HDI) and Sn(Oct)_2_ (purity > 95%) were purchased from Aladdin (Shanghai, China). Other reagents, including ethyl ether, and ethyl acetate (EA) (Macklin, Jinan, China) were of AR grade.

### Synthesis of OCN–PEG–NCO

**Figure 1:**
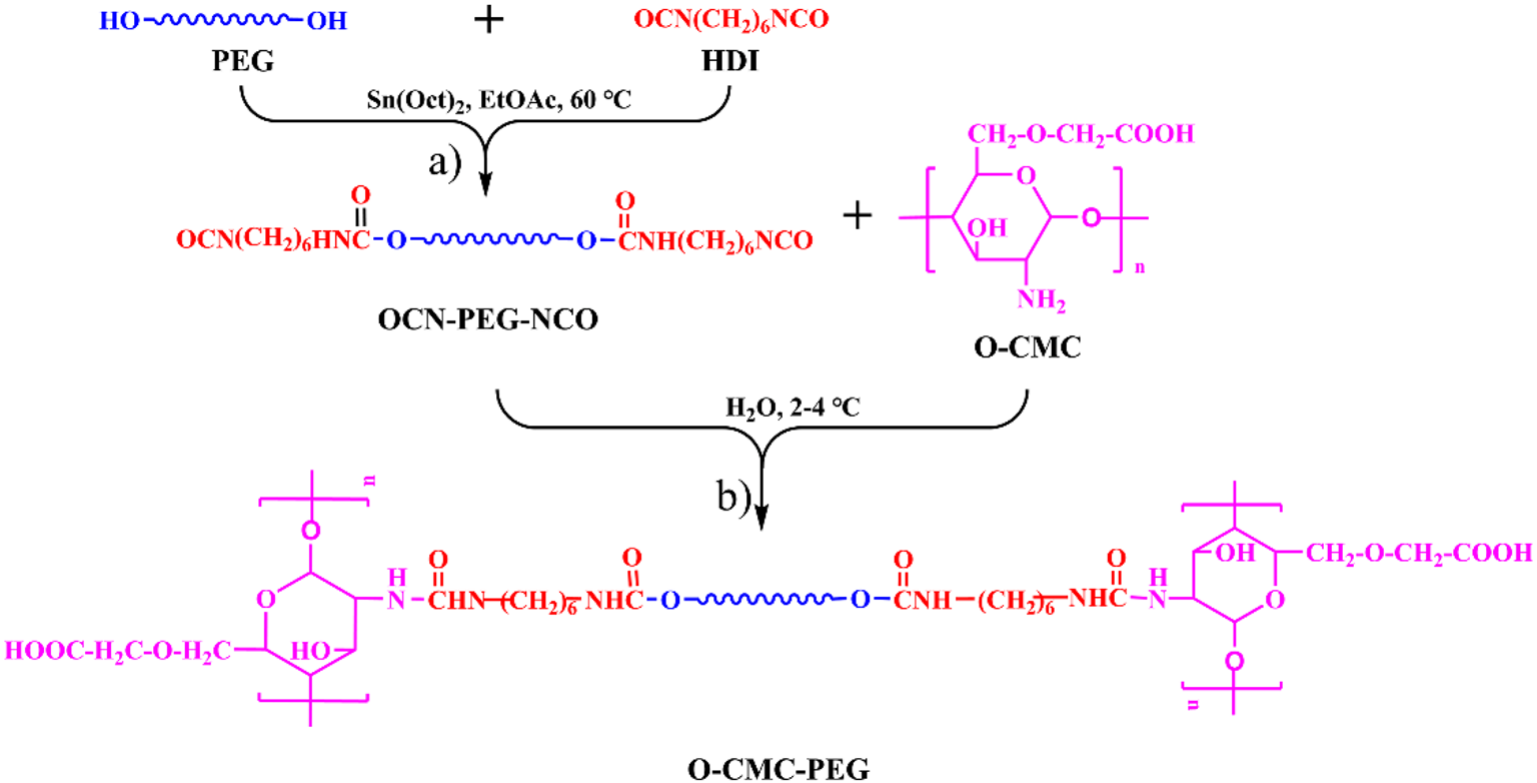
Synthetic processes of (**a**) OCN–PEG–NCO and (**b**) O-CMC–PEG.

The method for synthesis of OCN–PEG–NCO was described in the previous literature^37^ and the reaction scheme is illustrated in Fig. 1a. To summarize, the PEG (0.02 mol), HDI (0.12 mol), and Sn(Oct)_2_ (0.3 wt%) were dissolved in 15 mL of EA in the presence of dry N_2_. The reaction was conducted at 60 °C for 6 h. Subsequently, the resulting mixture was precipitated in cold diethyl ether to remove the catalyst and unreacted HDI. The purification process was executed three times. After drying under vacuum, the viscid OCN–PEG–NCO was obtained.

### Preparation of O-CMC–PEG Films

The reaction process can be observed in Fig. 1b. Typically, O-CMC was dissolved in deionized water under mechanical stirring at a temperature range of 2–4 °C to obtain a homogenous solution (0.1 g/mL). Then, the aqueous solution of OCN–PEG–NCO (0.2 g/mL) was introduced into the system. After rapid stirring, the mixture was gently transferred into a Teflon mold at the same temperature. The reaction was carried out in the mold at room temperature until the vanishing of –NCO absorption in the FT-IR spectrum (~ 2267 cm^−1^). Removal of solvent was achieved by volatilizing at 60 °C, resulting in the formation of O-CMC–PEG films (thickness: 0.10 ± 0.01 mm) which were thoroughly dried under vacuum. The basic formulations for O-CMC–PEG are provided in Table 1. The composite films with varying PEG content were labeled as O-CMC–PEG_−X%_ (where X% represents the weight content of PEG in the films).

**Table 1:** Basic formulations of O-CMC–PEG. (n_−NH2_/n_−NCO_: mole ratio of –NH_2_ and –NCO).

Film samples	O-CMC (g)	OCN–PEG–NCO (g)	n_−NH2_/n_−NCO_	PEG content (wt%)
O-CMC–PEG_−3%_	5.0	0.21	66:1	3.02
O-CMC–PEG_−6%_	5.0	0.44	32:1	6.05
O-CMC–PEG_−9%_	5.0	0.69	20:1	9.07
O-CMC–PEG_−12%_	5.0	0.96	14:1	12.06

### Measurements

FT-IR analyses were conducted on a NICOLET 6700 infrared spectrophotometer (USA) with a resolution of 4 cm^−1^. ^1^H NMR measurements were performed on a 400 MHz Bruker AVANCE II spectrometer (Germany). The microstructures of the film surfaces were observed with an SEM (Quanta 200, FEI, Holland).

The TGA 2950 instrument (TA, USA) was used to perform TGA with a ramp rate of 20 °C/min in the presence of N_2_. DSC analysis was conducted using a DSC2500 instrument (TA, USA) under N_2_ with a heating rate of 10 °C/min. Tensile testing was carried out on a UTM-0402 testing machine (Jijian Instruments, Chengde, China) following the standard testing procedure described in GB/T1040.3-2006.

Water vapor transmission rates (WVTR) were examined by the gravimetric method^38^. Typically, the sample films were cut into circular pieces and then were covered on the conical bottle containing anhydrous CaCl_2_, and placed in a relative humidity (RH = 30%, 50%, and 80%) incubator at 25 ± 1 °C. Then, the WVTR (g mm/ (m^2^·24 h)) of the samples was calculated by the change in the conical bottle quality after 24 h.

Water absorption (WA) measurement was performed in deionized water. The film samples were incubated in water at 25 ± 0.5 °C. At a predetermined period, the swollen samples were weighed after the excess water on the surface was gently wiped off with absorbent paper. The WA was calculated from the weight ratio of the swollen sample to the initial sample.

The degradation properties were executed according to the method in the published paper^39^. Briefly, the test samples were immersed in glass vials containing PBS (pH 7.4) and incubated at room temperature over 6 weeks. The mass loss percentages at specific time intervals were determined to evaluate the degradability. SEM was used to observe the surface morphologies of the lyophilized samples.

The antibacterial capacities of the film against *Escherichia coli* and *Staphylococcus aureus* were evaluated via an inhibition zone test^40^. In brief, the culture plate was first covered with a nutrient agar medium, and then the test bacteria (3 × 10^5^–5 × 10^5^ CFUs/mL) were spread on it. The sterilized film samples were positioned on the plate. After an incubation period of 24 h at room temperature, we measured the diameters of the inhibition zones to evaluate the antibacterial efficacy.

## Results and discussion

### Synthesis and characterization

**Figure 2:**
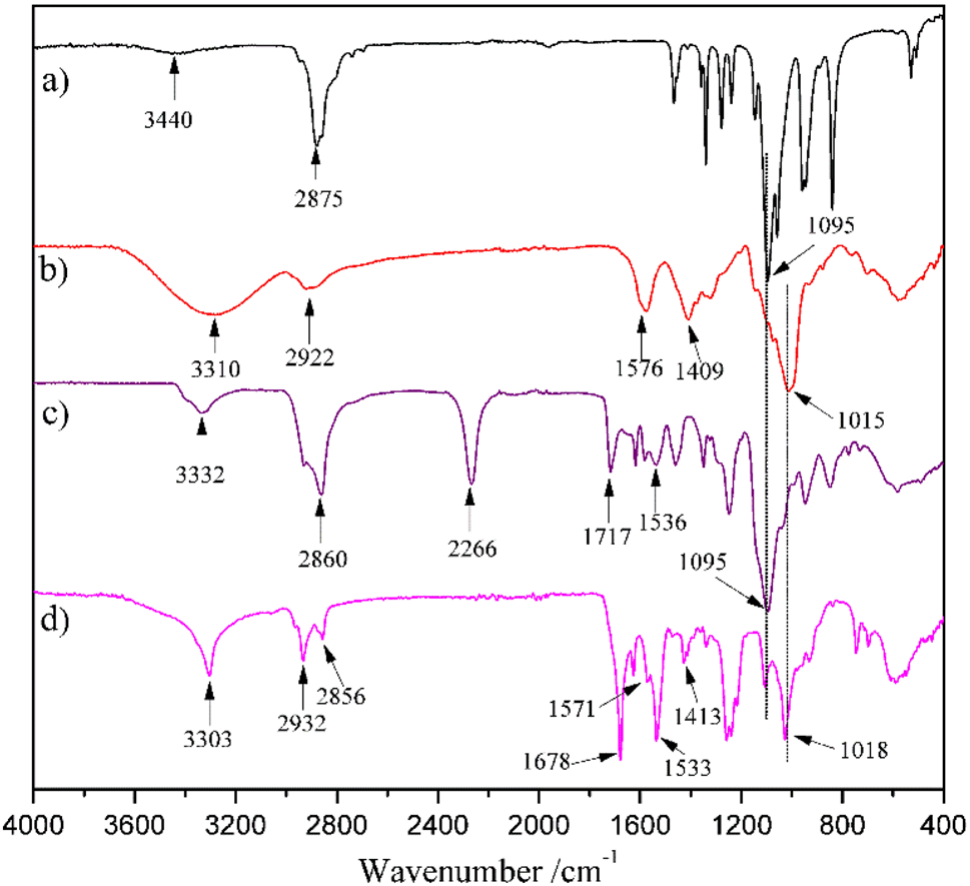
FT-IR spectra for (**a**) PEG, (**b**) O-CMC, (**c**) OCN–PEG–NCO, and (**d**) O-CMC–PEG_−6%_.

**Figure 3:**
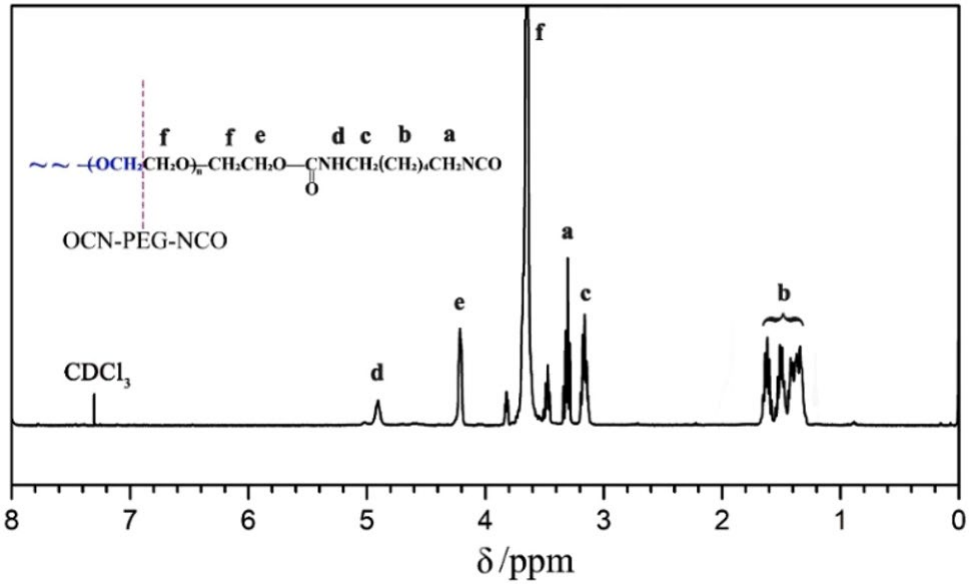
^1^H NMR spectrum of OCN–PEG–NCO.

The terminal –OH groups of PEG were end-capped with the –NCO groups to form the NCO–PEG–NCO linker without any byproducts. By using excess diisocyanate, the chain extension could be significantly limited. The chemical structure was analyzed by the FT-IR technique (Fig. 2c). In the spectrum, the broad absorption peak of the –OH groups in PEG (~ 3440 cm^−1^, Fig. 2a) disappeared^41^, accompanied by the presence of a new peak at 2266 cm^−1^ which vested in the typical absorption of –NCO groups. The peaks at 3332, 1717, and 1536 cm^−1^ should belong to the –NH–, amide, and C=O absorption in the newly formed urethane groups, respectively^42^. The analysis indicated that the terminal –OH groups of PEG were completely consumed and the –NCO groups were successfully introduced to the end of the PEG chain. Furthermore, the structure of OCN–PEG–NCO was confirmed by ^1^H NMR (Fig. 3). The chemical shifts at 4.85 ppm were attributed to the –N*H*– proton of urethane groups. The proton signals of urethane-linked methylene and repeat units (–OC*H*_2_C*H*_2_–) of PEG segments appeared at 4.22 and 3.63 ppm, respectively. Based on the molecular weight of PEG, the integral area of the proton peaks matched the chemical structure of OCN–PEG–NCO (Fig. 1), which further demonstrated no existence of a chain-extending byproduct.

For the reactivity of –NCO with –NH_2_ was much higher than that with –OH, the crosslinking reaction between O-CMC and OCN–PEG–NCO could be performed in water. A low temperature (2–4 °C) was used to reduce the reaction rate so that the reaction mixture could be easily transferred into the mold. The reaction endpoint could be monitored via the disappearance of the –NCO absorption in the FT-IR spectrum, and the composite films were produced by solvent evaporation. Due to the low solubility of the crosslinked O-CMC–PEG in water and organic solvents, their chemical structures were only characterized by FT-IR. The representative spectrum (O-CMC–PEG_−6%_) is shown in Fig. 2d. In the spectrum, the –NCO absorption at ~ 2266 cm^−1^ vanished completely, while two new strong peaks present at 1678 and 1533 cm^−1^ which vested in the H-bonded C=O and amide of the newly formed urea groups. The signals at 1571, 1413, and 1015 cm^−1^ belonged to the absorption of –COO^−^ asymmetric stretching, –COO^−^ symmetric stretching, and C–O–C vibration of saccharide ring from O-CMC (Fig. 2b), respectively. Meanwhile, the absorption peak belonging to ether C–O–C stretching at 1018 cm^−1^ of PEG (Fig. 2a) was retained. The broad absorption band at 3303 cm^−1^ was assigned to the –NH– absorption, which overlapped with the peak of –OH^43^. These results indicated that the terminal –NCO groups of OCN–PEG–NCO were completely reacted with –NH_2_ groups of O-CMC and the crosslinking structures linked by urea bonds were formed.

### Microstructure

**Figure 4:**
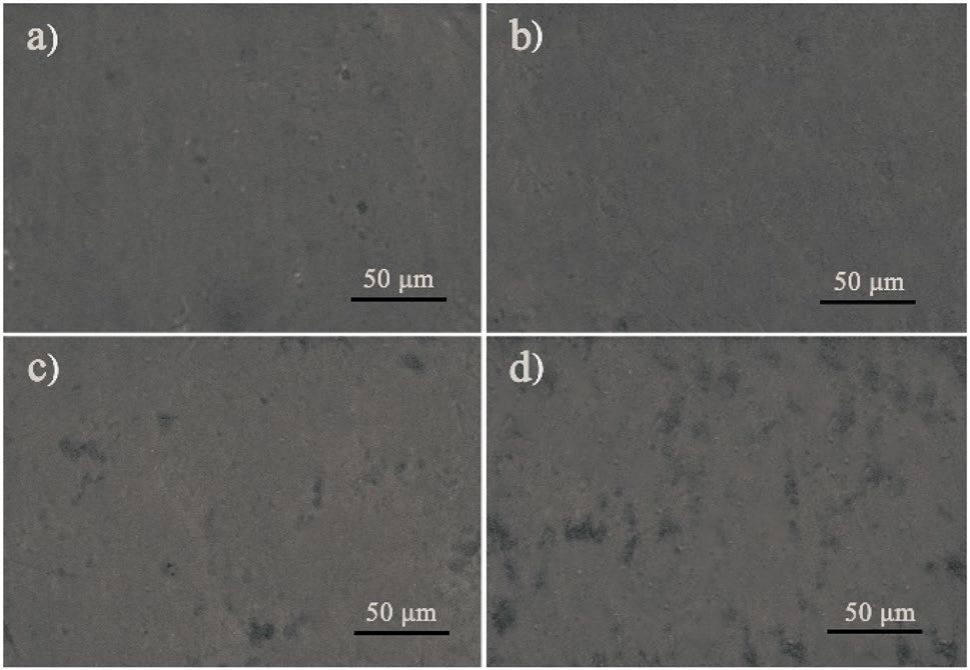
SEM images of (**a**) O-CMC–PEG_−3%_, (**b**) O-CMC–PEG_−6%_, (**c**) O-CMC–PEG_−9%_, and (**d**) O-CMC–PEG_−12%_.

The microstructure of a composite film was closely with its tensile properties. The SEM images of the O-CMC–PEG film surfaces are presented in Fig. 4. Compared with the microstructure of O-CMC–PEG_−3%_ (Fig. 4a), the film sample of O-CMC–PEG_−6%_ (Fig. 4b) exhibited a smoother surface and better compatibility between O-CMC and PEG, while the rough surface and obvious microphase separation appeared in the O-CMC–PEG_−9%_ and O-CMC–PEG_−12%_ films which had higher PEG content (Fig. 4c,d). Although the crosslinking by urea bonds and hydrogen bonds could enhance the interface adhesion and improve component compatibility^44^, the large polarity difference between O-CMC and PEG still resulted in two distinct O-CMC-rich and PEG-rich phases when the PEG content was high. Thus, to obtain fine component compatibility and favorable tensile performance, the PEG content in the crosslinked O-CMC–PEG composites should be controlled within a certain range.

### Thermal stability

**Figure 5:**
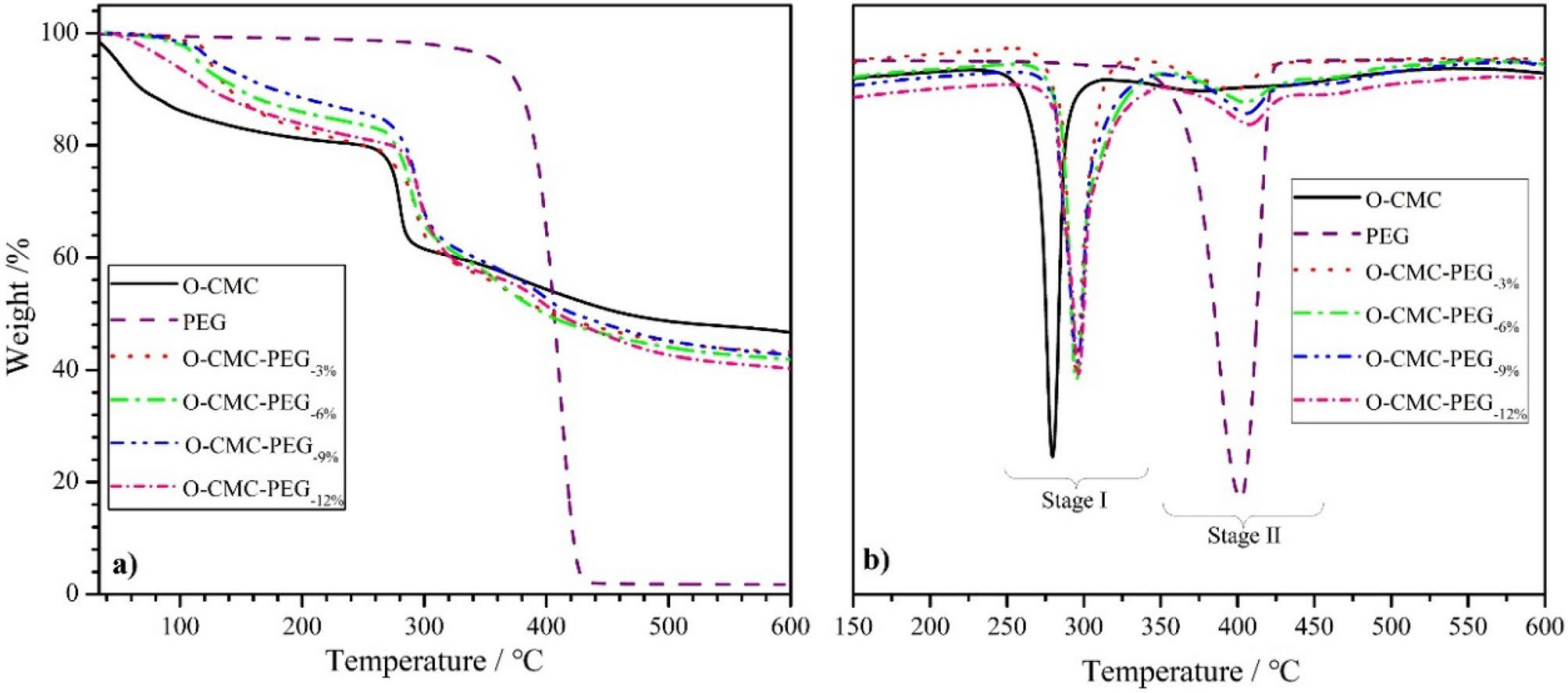
(**a**) TGA and (**b**) DTGA plots for O-CMC, PEG, and O-CMC–PEG films.

Figure 5 shows the TGA and DTGA plots for O-CMC, PEG, and O-CMC–PEG films with various PEG content, and the corresponding data obtained for the plots are summarized in Table 2. Only one-step weight loss ranging from 343 to 432 °C with a peak temperature (*T*_p_) of 402 °C was observed in the plot of PEG, while O-CMC exhibited 18.7% weight loss at 248–298 °C with a *T*_p_ of 279 °C and high residual weight (~ 46.8%) until the end of the test, which indicated that PEG had higher thermal stability in the relatively low-temperature region and lower thermal stability in the high-temperature region than O-CMC. After PEG was introduced into O-CMC to form crosslinked structures, the thermal degradation of prepared O-CMC–PEG films was mainly divided into two weight-loss stages with a small amount of moisture volatilization at ~ 100 °C. The first weight loss with the *T*_p−I_ at 254–258 °C belonged to the thermal dissociation of the O-CMC backbone. The second stage occurring at 397–407 °C (*T*_p−II_) showed less weight loss, which mainly originated from the dissociation of PEG segments. The weight loss was slightly lower than the theoretical PEG content in the composites due to the decomposition of CMC at this temperature range. With the increase of PEG content, the *T*_p_ values of the film samples present a gradual increase, demonstrating that the high crosslinking degree could improve their thermal stability to some extent. In addition, the residual weight of composite films was approximately 40% at the end of the tests, which belonged to the heat-resistant salt existing in O-CMC. The above analysis illustrated that the O-CMC–PEG films had high thermal stability and could be used over a wide range of temperatures.

**Table 2:** Thermal stability data of O-CMC, PEG, and O-CMC–PEG. (*T*_i_: initial decomposition temperature; *T*_p_: peak temperature).

Sample	Stage I			Stage II		
	*T*_i_ (°C)	*T*_p−I_ (°C)	Weight loss (%)	*T*_i_ (°C)	*T*_p−II_ (°C)	Weight loss (%)
O-CMC	248	279	18.7	–	–	–
PEG	–	–	–	343	402	94.6
O-CMC–PEG_−3%_	254	291	22.7	340	397	8.4
O-CMC–PEG_−6%_	256	293	22.5	343	404	9.2
O-CMC–PEG_−9%_	257	294	22.0	344	405	10.8
O-CMC–PEG_−12%_	258	295	21.2	351	407	13.1

### Thermal transition

**Figure 6:**
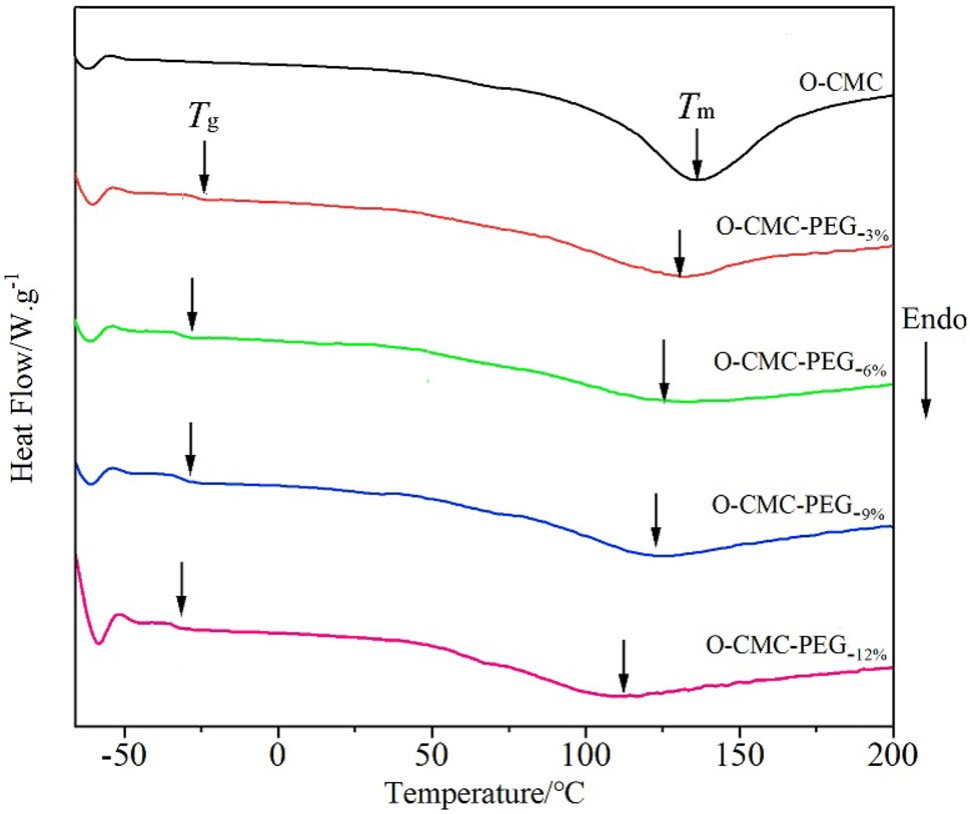
DSC for O-CMC powder and O-CMC–PEG films with different PEG content.

Figure 6 demonstrates the DSC profiles of O-CMC powder and O-CMC–PEG films containing varying amounts of PEG, and the thermal transition values extracted from these thermograms are summarized in Table 3. No evident glass transition temperature (*T*_g_) appeared in the O-CMC thermograms, which aligns with the findings of the previously published research^45^. On the other hand, a wide peak characterized by a melting temperature (*T*_m_) of 136 °C and enthalpy change (Δ*H*_m_) of 52.0 J/g emerged, indicating the melting transition of the crystalline phase and suggesting the semicrystalline characteristics of O-CMC. The O-CMC–PEG films exhibited a *T*_g_ at approximately − 26 °C, indicating that the composites were rubbery state at ambient temperature. Compared with O-CMC, a broader endothermic peak with a much lower Δ*H*_m_ value was present in the thermograms of O-CMC–PEG films, indicating a reduced crystallinity. When the PEG linker was introduced into O-CMC, the formed crosslinked structure restricted the chain motion and hindered the formation of crystalline domains^46^. Although the system was deemed partially miscible with immiscibility occurring in the amorphous phase^47^, it was noteworthy that the O-CMC–PEG_−6%_ film exhibited the lowest Δ*H*_m_ among the four samples, demonstrating superior component compatibility between O-CMC and PEG. This result was inconsistent with the microstructure analysis. Additionally, the *T*_m_ values of the endothermic peak steadily shifted to the lower temperature (Table 3) as the PEG content in the composites increased, which should be ascribed to the flexible nature of PEG.

**Table 3:** DSC values for O-CMC powder and O-CMC–PEG films.

Samples	*T*_m_ (°C)	*T*_g_ (°C)	Δ*H*_m_ (J g^−1^)
O-CMC	136	–	52.0
O-CMC–PEG_−3%_	136	− 25	16.9
O-CMC–PEG_−6%_	131	− 26	10.1
O-CMC–PEG_−9%_	126	− 26.5	15.7
O-CMC–PEG_−12%_	116	− 28	17.0

### Tensile properties

**Figure 7:**
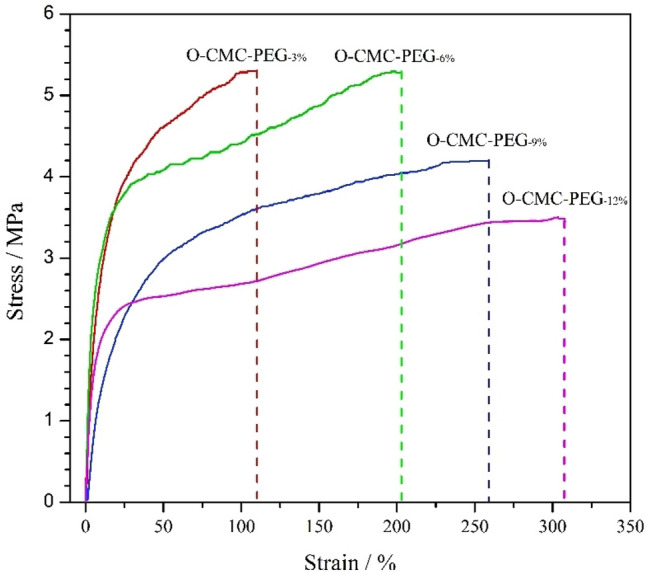
Representative tensile curves for O-CMC–PEG films.

High flexibility and toughness were the essential requirements for packaging materials. The tensile curves of O-CMC–PEG films encompassing various PEG content are portrayed in Fig. 7, and the corresponding tensile parameters are summarized in Table 4. A conspicuously smooth yield point was presented in the curves, signifying that all films demonstrated an elastic deformation within the initial short section and plastic deformation subsequently exceeding the yield point. The augmentation of PEG content in the composites led to an escalation in strain at break, whereas the ultimate stress diminishes. The phenomenon could be attributed to the high flexibility of the PEG soft segments which promoted the conversion of the brittle O-CMC into an elastomer^45^. As the papers reported, a high crosslinking degree was conducive to improving the ultimate stress, but the microphase separation produced by the high PEG content played the opposite trend on the strain at break and ultimate stress^41^. The film sample of O-CMC–PEG_−6%_ possessing superior component compatibility showed optimum tensile properties with strain at break of 202%, ultimate stress of 5.28 MPa, initial modulus of 30.5 MPa and fracture toughness of 8.89 MJ/m^3^, which was more suitable as a flexible packaging material.

**Table 4:** Tensile parameters for O-CMC–PEG films (n = 5).

Film samples	Strain at break (%)	Ultimate stress (MPa)	Initial modulus (MPa)	Fracture toughness (MJ/m^3^)
O-CMC–PEG_−3%_	109 ± 7	5.30 ± 0.6	30.8	4.75 ± 0.04
O-CMC–PEG_−6%_	202 ± 9	5.28 ± 0.5	30.5	8.89 ± 0.06
O-CMC–PEG_−9%_	259 ± 11	4.19 ± 0.4	23.6	8.91 ± 0.05
O-CMC–PEG_−12%_	308 ± 13	3.48 ± 0.3	22.5	8.98 ± 0.06

### Water absorption

**Figure 8:**
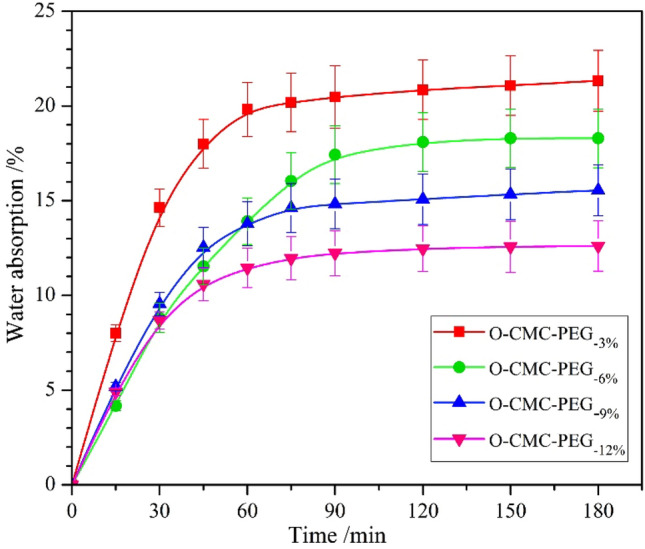
Water absorption of O-CMC–PEG films with various PEG content.

Water sensitivity, which was strongly linked with the application range of packaging, was evaluated by measuring their WA. Figure 8 displays the WA profile of O-CMC–PEG films after immersion in water at room temperature for 3 h. WA of the films exhibited rapid growth in the initial stage and achieved equilibrium with small differences. It was noticed that the O-CMC–PEG_−6%_ needed more time (~ 90 min) to reach the equilibrium WA than the other three samples (~ 60 min), which should be attributed to the homogeneous structure produced by crosslinking and high component compatibility. While with the increase of crosslinking degree in the composites (O-CMC–PEG_−9%_ and O-CMC–PEG_−12%_), the high PEG content resulted in microphase separation, which generated free space to allow water molecules to easily permeate the materials^48^. Based on values of equilibrium WA, it could be found the water sensitivity of the composites mainly depended on the crosslinking degree, that is, the crosslinking was an effective strategy to improve their water resistance. The relatively high equilibrium WA (> 12%) was due to the high hydrophilicity of PEG and O-CMC components. Thus, it could be deduced that O-CMC–PEG composites with moderate PEG content and high crosslinking degree would possess better water resistance by adopting OCN–PEG–NCO with lower molecular weight as the crosslinking agent.

### WVTR

**Figure 9:**
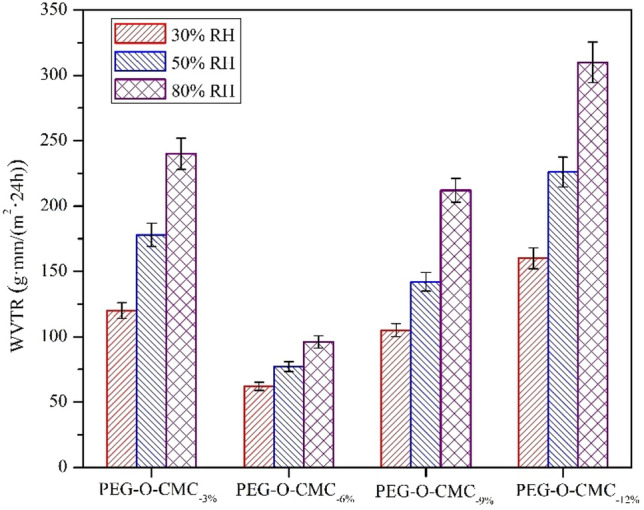
WVTR for O-CMC–PEG films at room temperature (*n* = 3).

Water vapor permeability was an important parameter for food packaging materials, and low WVTR could extend the shelf life of food^49^. Figure 9 displays the WVTR of the O-CMC–PEG films with various PEG content at RHs of 30%, 50% and 80%. For the samples with constant PEG content, the WVTR value exhibited a notable increase with the RH increasing from 30 to 80%, which was mainly attributed to the reduced humidity difference between the interior and exterior of the film. When the PEG content increased from 3% (O-CMC–PEG_−3%_) to 6% (O-CMC–PEG_−6%_), the WVTR values determined under the same RH showed a significant reduction. By enhancing the crosslinking degree, the miscibility between O-CMC and PEG was enhanced. As a result, the flexible PEG effectively occupied the gaps surrounding the O-CMC chains, thereby impeding the passage of water molecules and leading to a decreased WVTR. But for the O-CMC–PEG_−9%_ and O-CMC–PEG_−12%_ samples with relatively high crosslinking degrees, the WVTR presented a trend of sharp increase. The phenomenon should be due to the microphase separation caused by high PEG content in the samples, which offered convenient paths for the migration of water molecules. All the findings manifest that the WVTR of the composite films depended on both PEG content and crosslinking degree. The O-CMC–PEG_−6%_ with suitable PEG content and medium crosslinking degree possessed minimum WVTR (< 100 g mm/(m^2^ 24 h)) under different RH at 25 °C, meaning that the composite had good water vapor barrier performance and was suitable for food preservation, especially fruits and vegetables.

### Degradation properties

**Figure 10:**
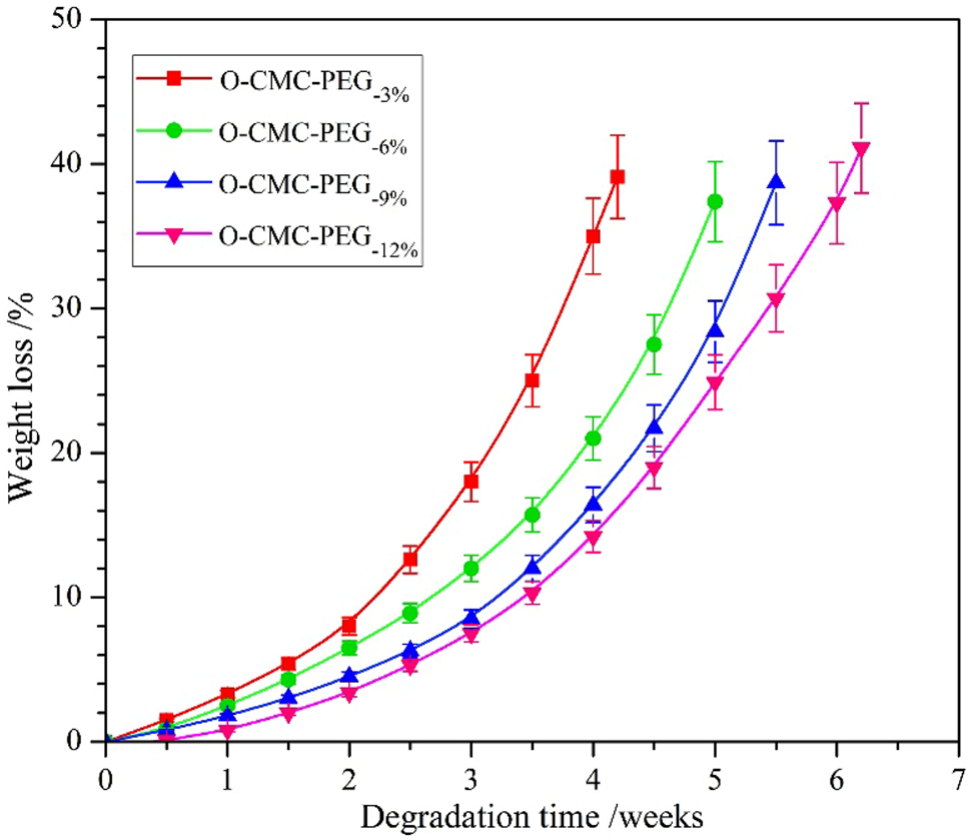
Degradation behaviors of O-CMC–PEG films (*n* = 3).

The appropriate degradability of packaging materials was necessary for the avoidance of white pollution to the environment. Figure 10 shows the weight loss-based degradation behaviors of O-CMC–PEG films in PBS (pH 7.4) at room temperature. All the films displayed a slow degradation rate with less than 8% weight loss in the initial two weeks, followed by accelerated weight loss until the fragmentation of the samples. After initial degradation, the film surface became porous, which improved the water molecules entering the films and increased the hydrolytic degradation rate. With the PEG content increasing from 3 to 12% (O-CMC–PEG_−3%_ ~ O-CMC–PEG_−12%_), the degradation rate of the composite film presented an obvious decrease. It was apparent that the degradation rate was mainly affected by the crosslinking degree in comparison with the PEG content and microphase separation. A similar tendency had been reported in crosslinked PVA/starch/citric acid composites^50^. From the results, it was inferred that the degradation rate of the O-CMC–PEG composites could be controlled by adjusting the crosslinking degree.

**Figure 11:**
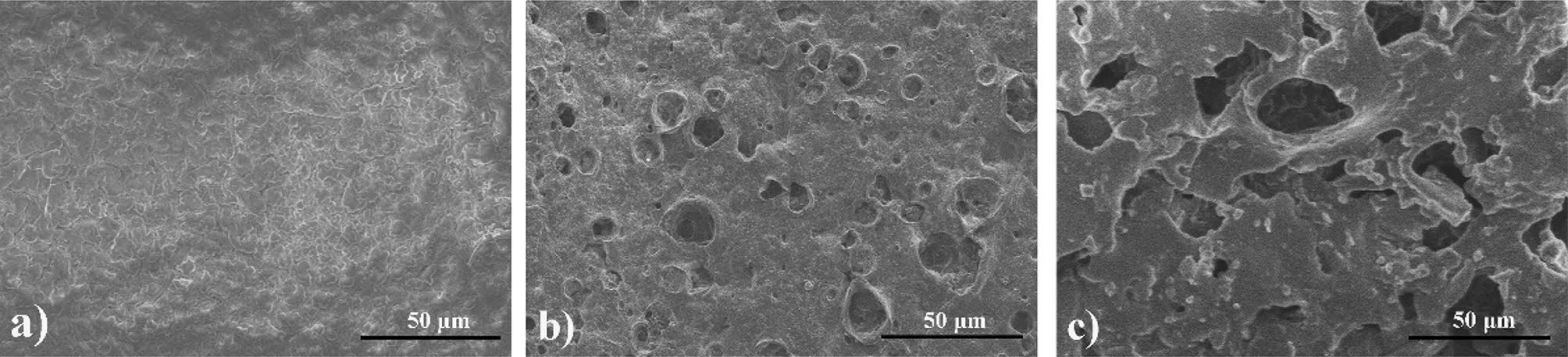
SEM images of O-CMC–PEG_−6%_ films after degradation for (**a**) 1, (**b**) 2.5, and (**c**) 4.5 weeks.

The morphological alterations on the film surface provided a direct indication of the degradation process. The lyophilized O-CMC–PEG_−6%_ films at various degradation stages were observed by SEM, as illustrated in Fig. 11. After one week of degradation, the initially smooth film surface exhibited roughness (Fig. 11a). As the degradation time progressed, some cavities of various sizes emerged on the surface (Fig. 11b). By the 4.5 weeks of degradation, many large cavities were visible (Fig. 11c), signifying substantial mass loss.

### Antibacterial activity

**Figure 12:**
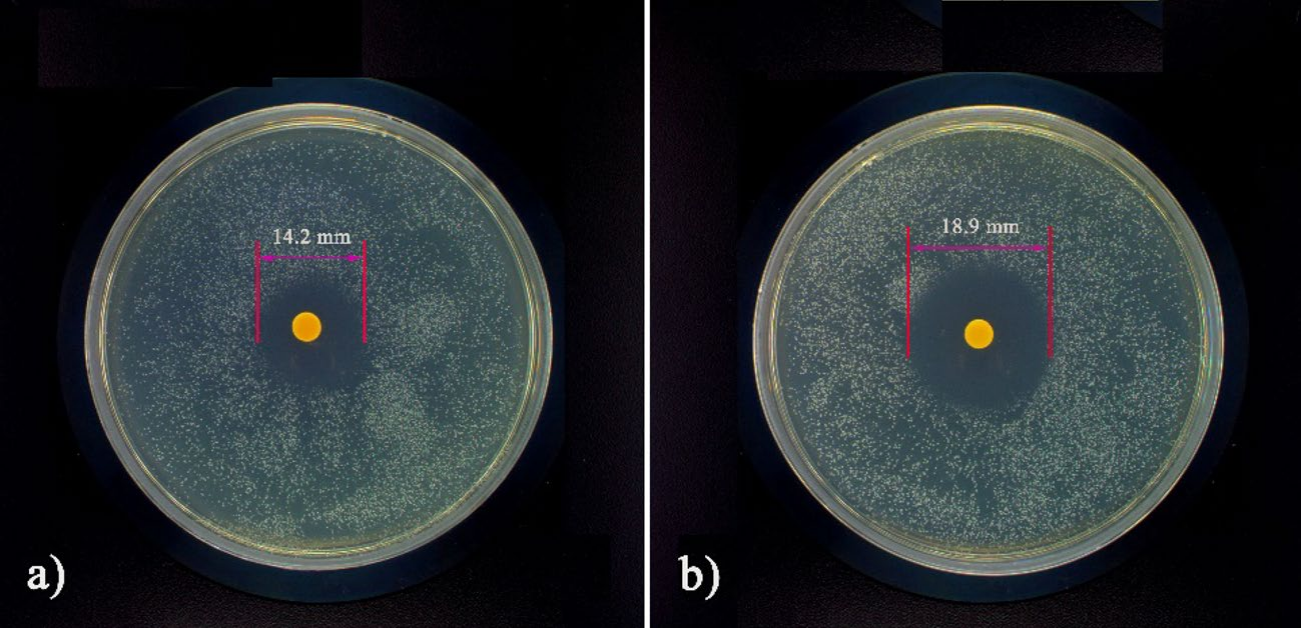
Antibacterial activities of O-CMC–PEG_−6%_ film against (**a**) *E. coli* and (**b**) *S. aureus*.

The antibacterial activities of O-CMC–PEG_−6%_ film were performed using inhibition zone assays against Gram-negative (GN) *E. coli* and Gram-positive (GP) *S. aureus*, and the findings are presented in Fig. 12. Following a 24-h incubation period at 25 °C the inhibition zone diameter for *E. coli* was determined to be 13.2 ± 0.4 mm, while *S. aureus* exhibited a diameter of 17.5 ± 0.3 mm, which demonstrated a good broad-spectrum antibacterial activity. This effectiveness could be ascribed to the presence of residual –NH_2_ (or –NH_3_^+^) groups within the O-CMC–PEG composites. These groups possessed the capability to bind to the negatively charged surface of bacterial cells through electrostatic adsorption, leading to membrane damage, intracellular nutrient leakage, and ultimately cell death^51^. The antibacterial activity was much higher against GP bacteria than GN bacteria, which was ascribed to the bilayer cell membrane of GN bacteria^52,53^. The positive broad-spectrum antibacterial activity manifested that the O-CMC–PEG_−6%_ films hold potential as antibacterial food packaging to prolong their shelf-life.

## Conclusion

In the work, a simple strategy was developed to prepare the PEG-crosslinked O-CMC (O-CMC–PEG) for food packaging. The condensation reaction between –NCO groups of PEG linker and –NH_2_ groups of O-CMC was readily accomplished in water under mild conditions to produce the crosslinked structure linked by stable urea bonds. Extensive research was conducted to investigate the impact of the PEG content (or crosslinking degree) on the physicochemical characteristics of the casted O-CMC–PEG films. The findings illuminated that crosslinking and components compatibility could improve their tensile features and water vapor barrier performance, while high PEG content showed the inverse effects due to the microphase separation. In comparison with the PEG content and components compatibility, in vitro degradation rate and equilibrium WA mainly depended on the crosslinking degree in the composites. Furthermore, the films exhibited good antibacterial capacities against *E. coli* and *S. aureus* due to the residual –NH_2_ groups of O-CMC. When the PEG content was 6% (medium crosslinking degree), the prepared O-CMC–PEG_−6%_ film possessed optimal tensile features (strain at break: 202%; ultimate stress: 5.28 MPa; initial modulus: 30.5 MPa; fracture toughness: 8.89 MJ/m^3^), relatively high water resistance (18.1% equilibrium WA), appropriate degradation rate (38.7% weight loss at 5.5 weeks degradation), low WVTR (77 g mm/(m^2^·24 h) at 50% RH) and fine broad-spectrum antibacterial capacity, manifesting a great potential for application in food packaging to extend the shelf life.

## Data Availability

The datasets used and/or analysed during the current study are available from the corresponding author on reasonable request.
